# Lesion topography shapes motor thresholds in brain tumor patients

**DOI:** 10.1016/j.nicl.2025.103924

**Published:** 2025-12-06

**Authors:** Alexia Stark, Kateryna Goloshchapova, Aldo Spolaore, Mykola Gorbachuk, Athanasios Gkampenis, Sophie Wang, Kathrin Machetanz, Marcos Tatagiba, Georgios Naros

**Affiliations:** Department of Neurosurgery and Neurotechnology, Eberhard Karls University, Tuebingen, Germany

**Keywords:** Navigated transcranial magnetic stimulation, Resting motor threshold, Voxel-based lesion-symptom mapping, Cortical excitability, Motor cortex, Brain tumors, Functional mapping

## Abstract

**Background:**

Navigated transcranial magnetic stimulation (nTMS) has become a cornerstone in preoperative functional mapping for brain tumor patients. The resting motor threshold (RMT) derived from nTMS reflects motor cortex excitability and may be influenced by tumor-related and patient-specific factors. However, the specific contribution of tumor location within cortical motor networks to RMT remains insufficiently understood.

**Methods:**

In this prospective study, 223 patients with motor-eloquent brain tumors underwent nTMS-based motor mapping. Individual RMTs were determined using the Rossini-Rothwell method. Preoperative MRIs were normalized to MNI space, and tumor lesions were manually segmented. Voxel-based lesion-symptom mapping (VLSM) was performed to assess voxel-wise associations between tumor location and RMT. Multivariate regression identified clinical and anatomical predictors of RMT.

**Results:**

Lesions were predominantly located in the perirolandic region, involving the primary motor cortex (MC) as well as precentral (preMC) and postcentral (postMC) areas. Multivariate analysis revealed that postMC tumor location and age were significant negative predictors of RMT, while meningioma histology was a positive predictor. VLSM revealed that lesions in the postcentral gyrus, superior parietal lobule, and precuneus were associated with lower RMT (i.e., increased excitability), whereas lesions in the precentral gyrus, supplementary motor area (SMA), and dorsal premotor cortex (PMd) were associated with higher RMT (i.e., decreased excitability).

**Conclusion:**

Motor cortex excitability in brain tumor patients is shaped by the functional integrity of interconnected cortical hubs. Disruption of inhibitory (e.g., sensory cortex) or facilitatory (e.g., premotor cortex) inputs to MC can modulate excitability in opposing directions. The combined use of nTMS and VLSM enables a network-level understanding of tumor-induced excitability changes and supports individualized surgical planning based on lesion topography.

## Highlights

• Tumor location significantly modulates resting motor threshold in brain tumor patients.
• VLSM reveals opposing excitability effects from precentral vs. postcentral lesions.
• Postcentral tumors are linked to lower RMT, indicating increased cortical excitability.
• Precentral and premotor lesions are associated with higher RMT, suggesting disfacilitation.
• This is the first study combining nTMS and VLSM to map excitability in tumor patients.

## 1 Introduction

Navigated transcranial magnetic stimulation (nTMS) has become an essential tool in preoperative neurosurgical planning in recent years (Picht, 2014, Raffa et al., 2019, Raffa et al., 2020, Frey et al., 2014, Ille et al., 2018, Krieg et al., 2012, Krieg et al., 2013, Raffa et al., 2019, Raffa et al., 2019, Sollmann et al., 2017, Eibl et al., 2023, Sollmann et al., 2021). Particularly in patients with brain tumors located near eloquent cortical areas, nTMS enables precise functional mapping of the motor cortex (MC) (Picht, 2014, Frey et al., 2014, Krieg et al., 2012, Sollmann et al., 2017, Sollmann et al., 2017, Sollmann et al., 2020). By allowing the non-invasive identification of motor pathways, nTMS significantly contributes to individualized surgical planning. Additionally, nTMS motor mapping improves the extent of resection (Raffa et al., 2020, Krieg et al., 2013, Hendrix et al., 2021, Krieg et al., 2012) and reduces the risk for postoperative neurological deficits (Sollmann et al., 2017, Krieg et al., 2014).

A key parameter in nTMS-based motor mapping is the resting motor threshold (RMT), which reflects excitability of the MC. RMT varies among patients due to multiple physiological and pathological factors, including tumor characteristics, cortical reorganization, and individual patient-specific variables (Eibl et al., 2023, Sollmann et al., 2021, Lavrador et al., 2021, Pitcher et al., 2003, Sollmann et al., 2013, Säisänen et al., 2021, Veldema et al., 2017; 2017., Rozand et al., 2019, Sollmann et al., 2017). Despite considerable research, the determinants of motor threshold remain incompletely understood.

One factor that has been relatively underexplored is the precise anatomical location of the tumor. Insights from dual-site TMS studies have demonstrated that MC excitability is subject to reciprocal modulation by interconnected hubs within the cortical motor network (Heemels et al., 2024, Hehl et al., 2024, Groppa et al., 2012, Brown et al., 2019, Davis et al., 2022). Specifically, the postcentral (postMC) areas has been shown to exert a predominantly inhibitory influence on MC excitability (Brown et al., 2019), whereas premotor regions (preMC) tend to have a facilitatory effect (Groppa et al., 2012).

In the present study, we combined nTMS-based motor mapping with voxel-based lesion-symptom mapping (VLSM) to investigate how tumor location within these functionally connected cortical hubs affects MC excitability. VLSM is a statistical neuroimaging technique that correlates the presence of lesions at specific brain voxels with behavioral or physiological outcomes (such as the motor threshold) across a patient cohort (Karnath et al., 2018, Baldo et al., 2022). Unlike region-of-interest analyses, VLSM allows for unbiased, voxel-wise evaluation of structure–function relationships across the entire brain (Karnath et al., 2018, Baldo et al., 2022). This approach is particularly well suited to assess whether tumors in specific cortical regions are systematically associated with changes in RMT.

We hypothesize that tumors involving regions such as postMC areas may lead to disinhibition of the MC and consequently lower motor thresholds, whereas involvement of preMC areas may reduce excitability. This study aims to elucidate how local tumor anatomy contributes to altered cortical excitability in brain tumor patients. A deeper understanding of these relationships is expected to enhance the precision of nTMS-based motor mapping and improve individualized surgical planning in neuro-oncological patients.

## 2 Methods

### 2.1 Participants and clinical data

This prospective study included 223 consecutive patients scheduled for brain surgery of motor-eloquent lesions. As part of the standard preoperative workup, these patients underwent navigated transcranial magnetic (nTMS) based motor mapping. Clinical data was acquired from medical records including age at diagnosis, sex, histopathological findings, the presence of seizures, the intake of antiepileptic medication (AED) and the presence of motor deficits as defined by MRC (Medical Research Council) grade > 1. Histopathological diagnosis was reclassified into low-grade (LGG, i.e. WHO grade 1 and 2) and high-grade (HGG, i.e. WHO grade 3 and 4) glioma as well as extra-axial lesions (i.e., meningioma and metastasis). Two experienced neurosurgeons evaluated the preoperative MRI classifying tumor side (i.e., left and right), location (i.e., cortical, subcortical, precentral, central, postcentral) and the presence of peritumoral brain edema (PTBE). The study was approved by the local ethics committee of the Medical Faculty at the Eberhard Karls University Tübingen. All patients gave written informed consent. Patient characteristics are summarized in Table 1.

**Table 1:** Patients‘ clinical and imaging characteristics.

	**Total** **N = 223**	**postMC** **N = 40**	**preMC** **N = 88**	**MC** **N = 72**
**Demographics**				
Age (years)	53.0 ± 17.4	55.4 ± 15.5	52.9 ± 17.8	52.6 ± 17.6
Sex f:m	107:116	18:22	37:51	38:34
Tumor volume (cm^3^)	31.4 ± 43.1	32.4 ± 30.7	25.1 ± 30.4	33.2 ± 36.4
AED intake	122 (55 %)	16 (40 %)*	63 (72 %)*	31 (43 %)*
Levetiracetam	107 (48 %)	15 (38 %)	54 (61 %)	28 (39 %)
Motor deficit	103 (46 %)	13 (33 %)	40 (46 %)	41 (57 %)*
PTBE	169 (76 %)	34 (85 %)	64 (73 %)	49 (68 %)

**nTMS parameter**				
MT (%)	45.3 ± 15.0	38.8 ± 9.0*	45.1 ± 13.8	47.9 ± 16.6
HS-X coordinate (mm)	38.5 ± 6.8	38.7 ± 6.4	37.8 ± 7.3	39.6 ± 6.0
HS-Y coordinate (mm)	−11.1 ± 8.9	−7.9 ± 5.6*	−14.0 ± 9.7*	−8.8 ± 7.6*
HS-Z coordinate (mm)	65.6 ± 5.2	65.1 ± 5.0	66.6 ± 5.3*	64.6 ± 5.0*
Dist2tumor (mm)	20.9 ± 18.3	21.6 ± 14.2	21.0 ± 18.8	18.1 ± 18.5*

**Histology**				
HGG	88 (40 %)	14 (35 %)	37 (42 %)	23 (32 %)
LGG	48 (22 %)	9 (23 %)	21 (24 %)	13 (18 %)
metastasis	39 (18 %)	11 (28 %)	11 (13 %)	21 (30 %)
meningioma	48 (22 %)	6 (15 %)	19 (22 %)	15 (21 %)

**Tumor location**				
cortical	188 (84 %)	36 (90 %)	79 (90 %)	66 (92 %)
subcortical	33 (15 %)	4 (10 %)	9 (10 %)	6 (8 %)
precentral	88 (40 %)	−	−	−
central	72 (32 %)	−	−	−
postcentral n/c	40 (18 %)23 (10 %)	− −	− −	− −
Side of lesion (r:l)	117:106	18:22	53:35	33:39

**Abbreviations:** AED: Antiepileptic drug, Dist2Tum: Distance from tumor to motor hotspot, GBM: Glioblastoma, HGG: High-grade glioma, HS-X coordinate: X-axis coordinate of the motor hotspot in MNI space, HS-Y coordinate: Y-axis coordinate of the motor hotspot in MNI space, HS-Z coordinate: Z-axis coordinate of the motor hotspot in MNI space, LGG: Low-grade glioma, MC: motor cortex, RMT: resting motor threshold, n/c: Not classifiable, nTMS: Navigated transcranial magnetic stimulation, postMC: postcentral location, preMC: precentral locations, PTBE: Peritumoral brain edema.

### 2.2 Magnetic resonance imaging

All patients underwent preoperative anatomical MRI on a 1.5T scanner (Skyra/Prisma-fit/Aera, Siemens Healthineers, Erlangen, Germany) equipped with an 8-channel head coil. A contrast-enhanced T1-weighted MPRAGE sequence was utilized (with 1 mm isotropic resolution, TR/TE: 2300/2.29 ms). The resulting anatomical images were imported into the nTMS system (Nexstim Eximia, version 3.2.2, Helsinki, Finland) for cortical mapping.

### 2.3 Navigated transcranial magnetic stimulation

The cortical mapping procedure began by co-registering each participant’s T1-weighted anatomical MRI to their head, achieving a registration error below 2 mm (Kraus and Gharabaghi, 2015, Leão et al., 2020, Mathew et al., 2016, Kraus et al., 2016, Naros et al., 2022). Cortical mapping was then performed using navigated TMS with a biphasic figure‐8 coil (eXimia®, Nexstim, Helsinki, Finland). First, the “hotspot” was identified as the stimulus producing the largest motor-evoked potential (MEP) in the contralateral abductor pollicis brevis (APB). The resting motor threshold (RMT) was determined for the APB by the Rossini-Rothwell (R-R) relative-frequency method, i.e. the lowest stimulus intensity eliciting a MEP of more than 50 μV in at least 5 out of 10 trials. To avoid confounding effects from pre-stimulus EMG activation, patients were instructed to keep the target muscles fully relaxed throughout the mapping procedure and real-time EMG monitoring was used to ensure a silent baseline (<10–20 µV). The stimulation was delivered in two phases: the first with a posterior–anterior (PA) induced current and the second with an anterior–posterior (AP) current, with the electric field orientation (computed by the eXimia software based on the individual MRI) maintained perpendicular to the targeted sulcus. Next, the cortex was mapped at 110 % of the RMT, starting at the primary motor cortex and extending to include the primary somatosensory and premotor areas. When brain tumors obscured the typical gyral and sulcal anatomy, mapping continued until no further MEP responses were obtained. Stimulation sites were visualized on the cortical surface at a depth of 25–30 mm, and their coordinates were automatically saved by the eXimia system.

### 2.4 Voxel-based lesion symptom mapping (VLSM).

**Fig. 1:**
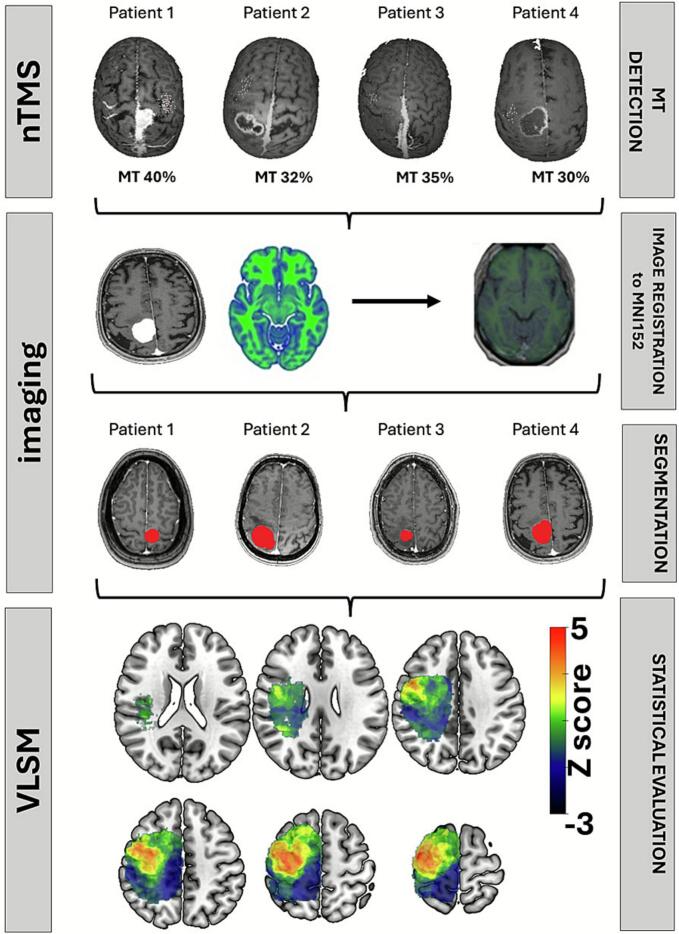
Illustration of the voxel-based lesion symptom mapping (VLSM) analysis pipeline. The present study integrates both nTMS and imaging information. All patients underwent nTMS-based motor mapping to determine the individual resting motor threshold (RMT). The preoperative T1-weighted MR images were registered and normalized to the MNI152 standard brain template. Tumor lesions were manually segmented and mirrored to the right hemisphere for standardization. Lesion masks from all patients were aggregated to identify voxels affected across the cohort. A voxel-wise linear regression was performed to assess the association between tumor presence at each voxel and individual RMT values, corrected for multiple comparisons using false discovery rate (FDR). The resulting statistical maps reveal brain regions where tumor localization significantly predicts changes in cortical excitability.

All Digital Imaging and Communications in Medicine (DICOM) format images were first converted to the Neuroimaging Informatics Technology Initiative (NIfTI) format by using *dcm2niix*. Statistical Parametric Mapping Software version 12 (SPM12, Institute of Neurology, University College London, London, UK; https://www.fil.ion.ucl.ac.uk/spm/docs/) and MATLAB (R2024a, MathWorks, Natick, MA, USA) was used to register and normalize patient’s MR images to a standard brain template (MNI152; Montreal Neurological Institute, McGill University, Montreal, Quebec, Canada) (Fig. 1A). Normalization was performed in SPM12 using unified segmentation and nonlinear warping. A lesion (cost-function) mask was applied to prevent the algorithm from using distorted tissue for spatial normalization. All normalized images were inspected visually, and manual corrections were performed when necessary to minimize mass-effect–related misregistration (Ashburner and Friston, 2005, Ripollés et al., 2012). The normalized image was resampled to a voxel size of 1x1x1mm. An experienced neurosurgeon manually outlined the tumor on the individual MR images using Mricro software (https://www.nitrc.org/projects/mricron). Lesion segmentation followed standard neuro-oncological criteria: enhancing tumors (high-grade glioma, metastasis, meningioma) were segmented on contrast-enhanced T1-weighted MPRAGE images, while non-enhancing low-grade gliomas were segmented based on T2-weighted and FLAIR hyperintensity. Peritumoral edema (PTBE) was explicitly excluded from the lesion mask (Ellingson et al., 2015). The tumor volume was measured and the corresponding tumor mask saved for further analysis (Naros et al., 2022). All lesion masks were mirrored to the right hemisphere to avoid hemispheric sparsity and increase statistical power in the voxel-wise model. This approach is commonly used when the behavioral variable is not hemisphere specific. Although some earlier studies have reported hemispheric asymmetries in motor excitability (De Gennaro et al., 2004, Triggs et al., 1997), well-controlled investigations demonstrate that RMT is highly symmetrical between hemispheres in healthy individuals (Cotovio et al., 2022, Säisänen et al., 2008). Original (non-mirrored) lesion laterality was retained for clinical analyses. Voxel-based lesion symptom mapping (VLSM) was based on a univariable linear regression model and one-tail t-statistics as implemented in SPM12 (https://www.fil.ion.ucl.ac.uk/spm) and NiiStat (https://www.nitrc.org/projects/niistat). VLSM enables to evaluate the relationship of a predictor (i.e., tumor location) to a specific outcome (i.e., the motor threshold) at individual voxels (i.e., voxel-vice) (Bates et al., 2003)(Fig. 1). Voxels with signal were included in the analysis, resulting in 218,825 analyzed voxels. Multiple comparison correction was based on the FDR technique (Benjamini and Hochberg, 1995, Mirman et al., 2018). Significant voxels were identified using a false discovery rate (FDR)–corrected threshold of q < 0.05 (voxel-wise).

### 2.5 Statistics

Statistical analyses were conducted using IBM SPSS Statistics for Windows (Version 25.0, Armonk, NY: IBM Corp.) alongside custom MATLAB scripts incorporating MATLAB's Statistics Toolbox. For comparison of categorical data, we used a chi-squared test (X2 test). Group comparisons of metric variables were based on non-parametric Mann-Whitney-U-Tests. p-values < 0.05 were considered significant. Results are shown as mean ± standard deviation (SD).

## 3 Results

### 3.1 Patient characteristics

A total of 223 consecutive patients with motor-eloquent brain lesions were included in the study. Pathologies comprised high-grade (88/223, 40 %) and low-grade glioma (28/223, 22 %) as well as metastasis (39/223, 18 %) and meningiomas (48/223, 22 %). Preoperative motor deficits were present in 103/223 (46 %) of patients at the time of nTMS mapping. A considerable proportion of patients (122/223, 55 %) were on antiepileptic medication (AED), most commonly Levetiracetam (107/122, 88 %). These results are summarized in Table 1.

### 3.2 Lesion characteristics

**Fig. 2:**
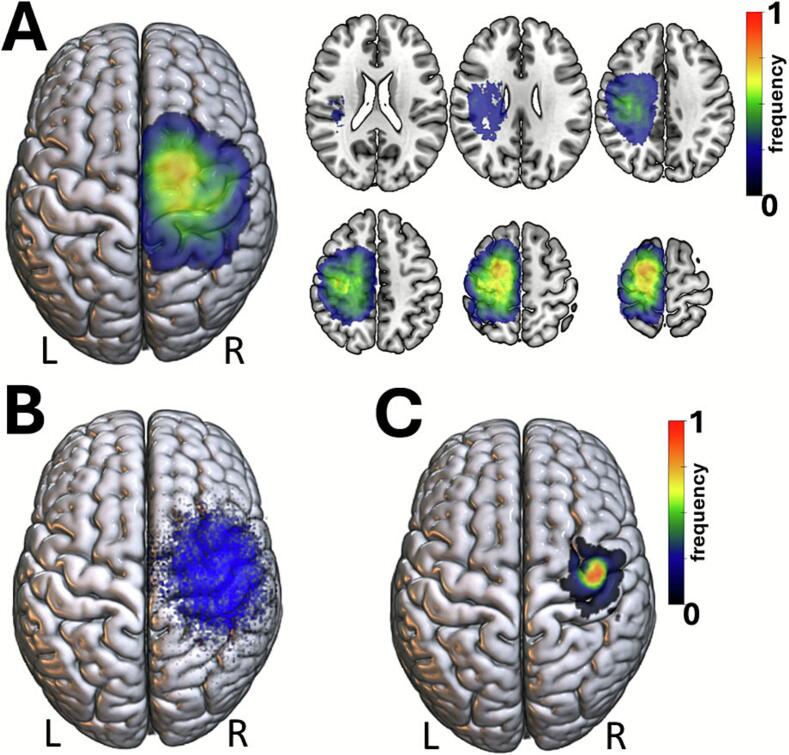
Spatial distribution of lesions and motor mapping results. (A) Aggregated lesion overlay showing the distribution of tumor locations across the cohort (n = 223) after normalization to MNI space. Most lesions were localized to the perirolandic cortex, involving precentral, central, and postcentral regions. No lesions affected subcortical motor pathways such as the internal capsule or basal ganglia. (B) Cortical mapping using navigated transcranial magnetic stimulation (nTMS) identified motor hotspots (HS) across patients, primarily located at the anatomical hand knob of the precentral gyrus. (C) Mean MNI coordinates of motor hotspots (X: 38.5 ± 6.8 mm; Y: –11.1 ± 8.9 mm; Z: 65.6 ± 5.2 mm) are visualized on the cortical surface.

After each individual preoperative MR images were registered to the MNI152 template space, tumor masks were aggregated to calculate the spatial distribution of all lesions. In the present cohort most lesions projected to the perirolandic cortex. There were no lesions affecting the basal ganglia and the posterior crus of the internal capsule (Fig. 2A). 40 patients (18 %) had tumors involving postcentral areas (postMC), 88 patients (40 %) precentral areas (preMC), and 72 patients (32 %) the motor cortex (MC). The topography of 23 patients (10 %) could not be definitively classified. The mean tumor volume was 31.4 ± 43.1 cm^3^ with 76 % showing a PTBE (Table 1). There were no significant group differences in tumor volume or the occurrence of PTBE. Notably, patients with preMC tumors showed a higher rate of AED use (72 %) compared to the remaining cohort (χ^2^ = 16.72, p < 0.001). The MC group had the highest rate of preoperative motor deficits (57 %, χ^2^ = 4.95, p = 0.026). These results are summarized in Table 1.

### 3.3 Results of the nTMS motor mapping

nTMS motor mapping covered the entire perirolandic cortex to detect the motor hotspot (Fig. 2B). The motor hotspot was located on the precentral gyrus at the area of the anatomical hand-knob in all patients (MNI coordinates X: 38.5 ± 6.8 mm, Y: −11.1 ± 8.9 mm, Z: 65.6 ± 5.2 mm) (Fig. 2C). PreMC lesions resulted in a slight but significant posterior shift of the motor hotspot (Table 1). The mean distance to the motor hotspot to the tumor (Dist2Tum) was 20.9 ± 18.3 mm with a significant reduction for the MC group (18.1 ± 18.5 mm, H = −2.05, p = 0.040). The individual resting motor threshold (RMT) was identified by the Rossini-Rothwell (R-R) relative-frequency method. The mean RMT for the overall cohort was 45.3 ± 15.0 %. In the postMC group, RMT was significantly lower compared to the remaining cohort (38.7 ± 9.0 %; H = 10.304, p = 0.001). There was no correlation between the Dist2Tum and the RMT (r = −0.078, R^2^ = 0.010, p = 0.147).

### 3.4 Predictors of resting motor thresholds (RMT)

A multivariate stepwise linear regression was conducted to examine the relationship between demographic (i.e., age, sex), clinical (i.e., AED intake, motor deficits), and tumor-related (i.e., volume, PTBE, Dist2Tum, HGG, LGG, metastasis, meningioma, subcortical/preMC/MC/postMC location) variables and RMT. After three iterations, a significant model (R = 0.277; F = 5.71, p < 0.001) was detected with postMC location and patient age as significant negative predictors and meningioma histology as positive predictor of RMT. All other variable were excluded from the model (Table 2). These findings indicates that tumor location has a major effect on RMT.

**Table 2:** Multivariate stepwise linear regression of RMT predictors.

	**Variables**	**β**	**T**	**p**
**included**	**postMC location**	−0.175	−2.607	0.010
	**meningioma**	0.170	2.467	0.014
	**age**	−0.150	−2.169	0.031

**excluded**	**LGG**	−0.018	−0.243	0.808
	**HGG**	−0.016	−0.213	0.831
	**metastasis**	0.037	0.515	0.607
	**PTBE**	0.056	0.785	0.433
	**subcortical location**	0.055	0.799	0.425
	**PreMC location**	−0.037	−0.510	0.611
	**MC location**	−0.028	−0.400	0.690
	**AED**	0.027	0.394	0.694
	**tumor volume**	0.055	0.796	0.427
	**Dist2Tum**	−0.098	−1.465	0.144

**Abbreviations:** AED: Antiepileptic drug, Dist2Tum: Distance from tumor to motor hotspot, HGG: High-grade glioma, LGG: Low-grade glioma, MC: Central motor cortex region, postMC: postcentral location, preMC: precentral location, PTBE: Peritumoral brain edema.

### 3.5 Tumor location and motor threshold

**Fig. 3:**
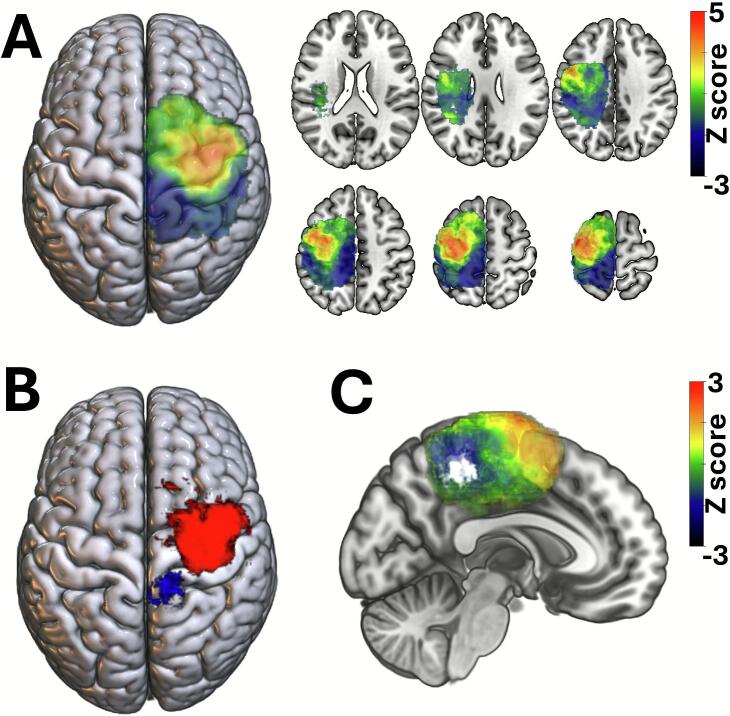
Voxel-based lesion-symptom mapping (VLSM) reveals anatomical predictors of resting motor threshold. (A) Lesions in the precentral gyrus, superior frontal gyrus (i.e., supplementary motor area, SMA), and medial frontal gyrus (i.e., dorsal premotor cortex, PMd) were significantly associated with increased resting motor threshold (RMT), indicating reduced cortical excitability. In contrast, lesions located posterior to the primary motor cortex showed a significant negative association with RMT, suggesting increased excitability due to disinhibition of the primary motor cortex. (B) These clusters survived FDR-based multiple comparisons correction. Significant voxels are highlighted in red (i.e., positive predictor) and blue (i.e., negative preditor). (C) The negative cluster involved the precuneus (AAL coordinates X: 9, Y: −56, Z: 44) supporting the involvement of this network hub in the modulation of motor cortex excitability. Voxels surviving FDR-based multiple comparisons correction are highlighted in white. (For interpretation of the references to colour in this figure legend, the reader is referred to the web version of this article.)

To further investigate the relationship between tumor location and its impact on RMT, we conducted a voxel-based lesion-symptom mapping (VLSM) analysis. This analysis revealed a positive association between tumors located in the MC and preMC regions—including the superior frontal gyrus and medial frontal gyrus— and increased RMT values, indicating reduced MC excitability. Functionally, this cluster extended to the supplementary motor area (SMA) and dorsal premotor area (PMd) (Fig. 3A,B). In contrast, tumors located in the postMC regions, such as the primary somatosensory cortex (S1) and superior parietal cortex, were negatively associated with RMT, suggesting increased MC excitability. Specifically, lesions involving the precuneus (AAL coordinates X: 9, Y: −56, Z: 44) were linked to lower RMT values (Fig. 3C).

## 4 Discussion

In this large prospective study of 223 patients with motor-eloquent brain tumors, resting motor threshold (RMT) was significantly influenced by tumor location, age, and tumor histology. Notably, tumors involving postcentral areas (postMC) were associated with lower RMTs, suggesting increased motor cortex (MC) excitability, whereas lesions in the precentral (preMC) regions and MC were linked to higher RMTs. Voxel-based lesion-symptom mapping (VLSM) confirmed these associations, highlighting the modulatory role of tumor-adjacent cortical areas. These findings indicate that local tumor anatomy and pathology significantly modulate cortical excitability as measured by nTMS.

In the present study, patient age was a major negative predictor indicating that higher age correlates with lower RMT(Eibl et al., 2023, Pitcher et al., 2003, Shibuya et al., 2016). This relationship may be attributed to structural disinhibition and compensatory neuroplasticity. Age-related loss of GABAergic inhibitory interneurons can lead to cortical hyperexcitability, which is further exacerbated by tumor-induced disruption of corticocortical networks (Sollmann et al., 2017, Ward, 2006). Moreover, older patients may recruit broader or alternative motor networks to preserve function, resulting in relatively lower RMT during stimulation (Taube and Lauber, 2025). Meningioma histology was significantly associated with higher RMT values, likely reflecting the mass effect and displacing nature of these extra-axial tumors, which may reduce the effectiveness of nTMS in stimulating the underlying MC (Raffa et al., 2020, Eibl et al., 2023, Sollmann et al., 2017). Contrary to many previous studies, glioma histology did not emerge as a primary predictor of RMT in our cohort (Eibl et al., 2023, Lavrador et al., 2021, Sollmann et al., 2017, Bates et al., 2003, Benjamini and Hochberg, 1995, Mirman et al., 2018). One possible explanation is that tumor location exerted a stronger influence on cortical excitability and may have masked histological effects. In line, previous research has shown that spatial proximity to motor-eloquent cortex is often more predictive of RMT than tumor type alone (Eibl et al., 2023).

The key finding of the present study is that tumor location has a major effect on RMT. Insights from dual-site TMS(dsTMS) studies have demonstrated that MC excitability is subject to reciprocal modulation by interconnected hubs within the cortical motor network (Heemels et al., 2024, Hehl et al., 2024, Groppa et al., 2012, Brown et al., 2019, Davis et al., 2022). The dorsal premotor cortex (PMd) and supplementary motor area (SMA) play a critical role in modulating excitability of the primary motor cortex (M1) through direct cortico-cortical projections exerting a short-latency facilitatory influence (Groppa et al., 2012). Similarly, the primary somatosensory cortex (S1) modulates M1 excitability, likely through sensorimotor integration pathways. Dual-site TMS paradigms probing S1–M1 interactions have demonstrated an inhibition depending on timing and motor task (Brown et al., 2019, Davis et al., 2022). These modulatory effects appear to be mediated, at least in part, by GABAergic inhibitory interneurons, as evidenced by the induction of short-interval intracortical inhibition (Hehl et al., 2024). Furthermore, the precuneus is part of the default mode network and has been recently associated with motor planning, particularly in tasks involving motor imagery, motor intention, and visuomotor integration. Interaction between the precuneus and M1 have been shown to modulate cortical excitability (Bonnì et al., 2013, Chao et al., 2015), a phenomenon that is currently explored for TMS treatment of Alzheimer’s disease (Koch et al., 2022). In summary, these findings highlight the functional relevance of inputs of secondary motor hubs in shaping MC excitability and support the notion that disruption of these networks (e.g., by tumors) can have significant effects on RMT. These functional network interactions are supported by the present finding. In our cohort, affection of PMd and SMA by brain tumors resulted in a disfascilitation of MC, i.e. a decrease of cortical excitability. In contrast, affection of the sensory cortex and precuneus resulted in a disinhibition of MC, i.e. an increase of cortical excitability. It is expected that destructive lesions (e.g., high-grade glioma) might have a stronger effect than infiltrating (e.g., low-grade glioma) and extra-axial tumors (e.g., meningioma and metastasis) lesions (Krieg et al., 2012). At the same time, it is expected that GABAergic medication (e.g., AED) will interfere with this phenomenon. Contrary, AED intake did not significantly influence RMT in the present study, despite previous reports (Eibl et al., 2023, Sollmann et al., 2017). However, most patients in the present cohort were taking levetiracetam, whose mechanism of action is not mediated by purely GABAergic effects but rather involves modulation of synaptic vesicle protein 2A and indirect regulation of neuronal excitability (Contreras-García et al., 2022).

To the best of our knowledge, this is the first study combining VLSM techniques and nTMS information. While nTMS provides individualized, non-invasive mapping of motor function, VLSM enables an unbiased, voxel-wise statistical analysis of lesion-behavior relationships across the entire cortex. This integrative approach enhances our understanding of how specific tumor locations modulate nTMS effects and allows the identification of functionally critical network hubs beyond the primary motor cortex. In the future, combining VLSM with longitudinal nTMS data may help track functional reorganization over time or under therapeutic interventions. Furthermore, incorporating connectomic and functional imaging data could extend this framework toward a multimodal network-based model of brain function, ultimately improving individualized risk stratification and surgical planning.

### 4.1 Limitations

Several limitations should be acknowledged. First, while our study included a large patient cohort, the heterogeneity of tumor types and locations may have introduced variability that could not be entirely accounted for. Pooling extra-axial and infiltrative tumors is a limitation, as their impact on cortical excitability may differ. Although sample size prevented fully stratified VLSM analyses, we acknowledge that tumor biology likely modulates the strength of excitability changes In addition, hemispheric mirroring may obscure potential lateralized functional differences between left and right sensorimotor cortices. Future studies with more homogeneous subgroups may provide a clearer understanding of the specific effects of individual tumor characteristics on RMT. Second, the timing between the AED intake and nTMS mapping was not systematically recorded, which limits our ability to assess the pharmacological influence on RMT. A more controlled, prospective study design would allow for a more detailed analysis of AED impact on RMT and the interaction with tumor location.

## 5 Conclusions

This study demonstrates that the resting motor threshold is not merely a local measure of primary motor cortex excitability, but reflects the functional integrity of a broader cortical network involving sensory, premotor, and associative areas. By combining nTMS with voxel-based lesion-symptom mapping, we provide novel insights into how specific tumor locations modulate cortical excitability in opposing directions. These findings might explain the part of the controversy of RMT literature and underline the importance of considering lesion topology within functional motor networks.

## Patient consent

All participants gave written informed consent.

## CRediT authorship contribution statement

**Alexia Stark:** Writing – review & editing, Writing – original draft, Formal analysis, Data curation. **Kataryna Goloshchapova:** Writing – review & editing, Formal analysis, Data curation. **Aldo Spolaore:** Writing – review & editing, Formal analysis, Data curation. **Mykola Gorbachuk:** Writing – review & editing, Formal analysis, Data curation. **Athanasios Gkampenis:** Writing – review & editing, Formal analysis, Data curation. **Sophie Wang:** Writing – review & editing, Formal analysis, Data curation. **Kathrin Machetanz:** . **Marcos Tatagiba:** Writing – review & editing, Validation, Supervision, Project administration. **Georgios Naros:** Writing – review & editing, Writing – original draft, Visualization, Validation, Supervision, Software, Resources, Project administration, Methodology, Investigation, Funding acquisition, Formal analysis, Data curation, Conceptualization.

## Ethics approval

The study was approved by the local ethics committee of the Medical Faculty of the Eberhard Karls University Tuebingen.

## Funding

There was no external funding of this study.

## Declaration of competing interest

The authors declare that they have no known competing financial interests or personal relationships that could have appeared to influence the work reported in this paper.

## Data Availability

Data will be made available on request.

## Glossary

**AED**: Antiepileptic drug
**AP**: Anterior–posterior (current direction in TMS)
**APB**: Abductor pollicis brevis (muscle)
**DICOM**: Digital Imaging and Communications in Medicine
**Dist2Tum**: Distance from tumor to motor hotspot
**DMN**: Default mode network
**dsTMS**: Dual-site transcranial magnetic stimulation
**EMG**: Electromyography
**HGG**: High-grade glioma
**HS**: motor hotspot
**ISI**: Interstimulus interval
**LGG**: Low-grade glioma
**MC**: motor cortex
**MEP**: Motor-evoked potential
**M1**: Primary motor cortex
**MNI**: Montreal Neurological Institute
**MNI152**: Standard brain template from the Montreal Neurological Institute (152 subjects)
**MRI**: Magnetic resonance imaging
**MRC**: Medical Research Council (muscle strength grading scale)
**nTMS**: Navigated transcranial magnetic stimulation
**NifTI**: Neuroimaging Informatics Technology Initiative
**PA**: Posterior–anterior (current direction in TMS)
**PMd**: Dorsal premotor cortex
**postMC**: postcentral areas
**preMC**: precentral areas
**PTBE**: Peritumoral brain edema
**RMT**: Resting motor threshold
**ROI**: Region of interest
**S1**: Primary somatosensory cortex
**SMA**: Supplementary motor area
**SPM**: Statistical Parametric Mapping
**TE**: Echo time (in MRI)
**TMS**: Transcranial magnetic stimulation
**TR**: Repetition time (in MRI)
**VLSM**: Voxel-based lesion-symptom mapping.
